# Edible solid lipid nanoparticles (SLN) as carrier system for antioxidants of different lipophilicity

**DOI:** 10.1371/journal.pone.0171662

**Published:** 2017-02-13

**Authors:** Kathleen Oehlke, Diana Behsnilian, Esther Mayer-Miebach, Peter G. Weidler, Ralf Greiner

**Affiliations:** 1 Institute for Food Technology and Bioprocess Engineering, Max Rubner-Institut, Karlsruhe, Germany; 2 Karlsruhe Institute of Technology (KIT), Institute of Functional Interfaces, Division of oxidic and organic interfaces Hermann-von-Helmoltz-Platz 1, 76344 Eggenstein-Leopoldshafen, Germany; Universidad de Castilla-La Mancha, SPAIN

## Abstract

Ferulic acid (FA) and tocopherol (Toc) loaded solid lipid nanoparticles (SLN) were prepared by a hot homogenisation method. The particle size distribution, zeta potential and melting behaviour of the SLN as well as the stability, encapsulation efficiency and radical scavenging activity of FA and Toc in the SLN were analysed. The different formulations containing up to 2.8 mg g^−1^ of FA or Toc were stable during at least 15 weeks of storage at room temperature. Despite partial degradation and / or release of FA and Toc during storage, significant radical scavenging activity was maintained. DSC measurements and radical scavenging tests after different time periods revealed that the re-structuring of the lipid matrix was connected to the enhanced antioxidant activity of Toc but did not affect the activity of FA.

## Introduction

Solid lipid nanoparticles (SLN) have been investigated as carrier systems in pharmaceutical, cosmetic or food related applications. The potential use of SLN includes the encapsulation of bioactive compounds, antimicrobials or antioxidants [1]. The encapsulation of antioxidants in SLN prepared using food grade constituents could be beneficial from both a physiological and a technological point of view. SLN formulations yielding stable SLN have been widely reported in the literature, but mainly with a pharmaceutical background, hence also contained non-food grade materials. Such SLN were demonstrated to be physically stable up to three years [2]. The solid lipid matrix of SLN has initially been regarded to represent an effective physical barrier against unfavourable conditions resulting in an increased chemical stability of encapsulated compounds. However, it was reported that the chemical stability of encapsulated compounds depends on the composition of the lipid matrix and the emulsifiers used, as e.g. in the case of *β*-carotene [3, 4].

Encapsulation rates near 100% or payloads of 1.1% for lipophilic compounds like tocopherol [5], rosmarinic acid [6] or vitamin A [7] have been reported. For compounds with decreasing lipophilicity the encapsulation efficiency decreased, still remaining in the range of 20 to 26% of the dry matter [8], but values of up to 85% have been reported for the more hydrophilic ferulic acid [9]. The encapsulation rate is closely related to the composition of the SLN, i.e. the lipid matrix and the emulsifiers used to stabilise the SLN. The crystallinity of the lipid matrix is among the most important and most investigated properties of SLN.

Among others, the antioxidant or radical scavenging activity of the encapsulated compounds was investigated in several studies and was assessed in different test systems like rat brain microsomes [10], cell systems [8] and with different radical initiators like AAPH or t-BOOH [11] where significant radical scavenging activity was observed. In contrast, SLN encapsulated co-enzyme Q10 showed negligible reactivity against ABTS [12].

The radical scavenging activity of an encapsulated compound is largely affected by its location within the SLN. Comprehensive experiments with different spin probes or curcuminoids revealed that encapsulated compounds are mainly located at the surface of SLN and that their location depends on their lipophilicity and the composition of the emulsifier layer [13–16]. The surface of SLN comprises the emulsifiers, i.e. antioxidants may be buried in the emulsifier layer of SLN. The location within the emulsifier layer has been reported to determine the activity of antioxidants in micelles, emulsions and SLN [17, 18]. In SLN the solid lipid matrix undergoes polymorphic transitions directly after preparation and during storage of the SLN. Due to different packing orders of *α*, *β*′ and *β* polymorphs, polymorphic transitions have been found to affect the release or activity of the encapsulated compounds [18]. This may be regarded as shortcoming if a stable formulation with constant properties of prolonged periods of time is needed. However, the expulsion and / or slow and gradual release of the active compound can also be beneficial e.g. if the antioxidant activity of an encapsulated compound has to be maintained during extended storage periods.

Ferulic acid (FA) and tocopherol (Toc) are common phenolic antioxidants occurring naturally or being added to food. In the present study these compounds were selected as representative hydrophilic (FA) or lipophilic (Toc) model compounds for antioxidants relevant to food applications. Based on their different molecular structures and physico-chemical properties different encapsulation rates and interactions with SLN were expected.

The aim of this study was to investigate the encapsulation efficiency and the chemical and physical stability of FA and Toc loaded SLN and how these factors are related to the radical scavenging activity of the encapsulated antioxidants. Both time and concentration dependent effects were investigated. Hence, this study contributes to understanding the potential of SLN as delivery systems for antioxidants in food systems.

## Materials & methods

### Materials

Glyceryl tristearate (96%, technical grade, TS), polysorbate 20 (< 3% water, Tween20^®^), trans-ferulic acid (99%, FA), *α*-tocopherol (96%, Toc) and potassium nitrodisulfate (99%, Fremy’s salt) were purchased from Sigma Aldrich (Steinheim, Germany). Solvents of the highest analytical grade were obtained from Carl Roth, Karlsruhe, Germany. Sodium acetate trihydrate was obtained from Merck, Darmstadt. De-oiled soybean lecithin (Epikuron 100 P) and Ryoto sugar ester S1670 were kind gifts from Cargill, Hamburg, Germany and Harke Food Tech, Mühlheim, Germany, respectively. All chemicals were used as received without further purification. Ultrapure water (MilliQ, Millipore) was used throughout the experiments. Unless stated otherwise solutions and suspensions were prepared using a 0.2 M acetic acid/sodium acetate buffer solution, pH 5.0.

### Preparation of SLN

SLN were prepared by ultrasound assisted hot emulsification. For each batch of SLN 2.5 g glyceryl tristearate and 125 mg lecithin were mixed and melted by heating to 80°C under stirring. The mixture was kept at this temperature for additional 30 min to completely remove possible crystal memory. Afterwards 7.5 g of a 30.2 ml mL^−1^ hot aqueous solution of S1670 was added to the melt. The mixture was emulsified for 30 min by ultrasonic homogenisation using a sonicator (Sonopuls HD 3100, Bandelin electronic GmbH, Berlin, Germany) with a titanium tip (VS70T) applying an amplitude of 75% in pulsed mode with 0.5 s pulse length. During sonication the temperature was maintained at 80°C using a thermostatted water bath. The hot emulsion was mixed with an equal amount of an aqueous 8% Tween20^®^ solution and cooled within 1 min to 20°C under stirring in an ice bath. The final composition of the SLN suspension was: 5% glyceryl tristearate, 1.35% S1670, 0.25% lecithin and 4% Tween20^®^. All percentages are weight based. Toc or FA loaded SLN (Toc-SLN, FA-SLN) were prepared by dissolving the appropriate amount of Toc or FA in the hot emulsion before the Tween20^®^ solution was added. To exclude possible changes in particle size due to the addition of large amounts of antioxidants, the respective amounts of tristearate were replaced by accurately weighed amounts of FA or Toc. Final FA or Toc contents in the SLN were between 0.56 mg g^−1^ and 2.80 mg g^−1^ of dispersion. Samples were prepared in triplicates in a randomised order and stored at 25°C.

### Zeta potential and particle size distribution measurements

The zeta potential was measured by electrophoretic mobility of the particles using a ZetaSizer Nano ZS (Malvern Instruments, UK). Samples were diluted approx. 100 fold with ultrapure water resulting in a conductivity of 50 ± 5 μS cm^−1^. The same samples and instrument were used for the particle size distribution measurements by dynamic light scattering at 25°C. The backscattered light was collected at 173°. The data were evaluated based on the intensity weighted hydrodynamic diameter (z-average) and polydispersity index (PdI) calculated from the cumulants analysis. To obtain an insight in the possible presence of several particle populations number based particle size distributions were obtained from the intensity based particle size distributions using Mie theory. All measurements were performed in triplicate.

### Scanning electron microscopy

Morphological analysis of SLN suspensions was carried out using a QuantaTM 250 FEG-SEM (FEI, Brno, Czech Republic) equipped with an Everhart-Thornley detector and operated at 10 kV accelerating voltage. The SLN suspension was diluted 1000-fold with 0.2 M sodium acetate buffer (pH 5.0) and 5 μL was deposited onto silicon substrates and mounted onto aluminium stubs with double coated carbon conductive tabs.

### Extraction of FA or Toc from SLN

For the determination of the total FA or Toc content in the SLN suspension, the following extraction procedure was applied: 50 mg of the SLN suspension was dissolved in 1 mL of methanol. The solution was heated to 60°C for 30 min interrupted by three sonication steps in a sonication bath for 1 min each. Finally, the mixture was centrifuged (10621 *g*, 40°C) and the clear supernatant was transferred into a volumetric flask. A second similar extraction step was applied and the pooled supernatants were analysed by HPLC. The recovery (%) was calculated as $Rec\%=100\frac{C_{ex}}{C_{ini}}$ with *C*_*ex*_ being the concentration of extracted FA or Toc and *C*_*ini*_ being the initially added amount of the respective antioxidant. The recovered amount of antioxidants included both, encapsulated and non-encapsulated FA or Toc. All samples were extracted in duplicates yielding six individual values for each antioxidant and concentration.

### Determination of the encapsulation efficiency

The encapsulation efficiency (EE%) was determined by ultrafiltration using centrifugal ultrafiltration devices (VivaSpin 2, 300 kDa, Sartorius Stedim Biotech, Göttingen, Germany) that were pre-rinsed with buffer solution. The filtration units were filled with 1.2 mL of SLN suspension and then centrifuged for 15 min at 5000 *g* and 25°C. Filtrate and retentate were removed and the filtration steps were repeated for a total of 6 times. The membrane was equilibrated during the first three filtration steps from which the filtrate and retentate were discarded. The concentrations of FA or Toc in the filtrates of steps 4–6 were analysed separately by HPLC using the same methods as described below. Two independent ultrafiltration devices were used for each sample yielding a total of 18 individual values for each concentration and antioxidant. The encapsulated amount was calculated as the difference between the total content previously determined by extraction, *C*_*ex*_, and the concentration of FA or Toc in the filtrate, *C*_*filtrate*_: $EE\%=100\frac{C_{ex}-C_{filtrate}}{C_{ex}}$.

### HPLC analysis

FA was analysed on an Agilent 1100 system applying the following method: 10 *μ*L of each sample was injected onto the column (SynergiHydro C18, 4 *μ*m, 150 x 4.6 mm, Phenomenex). The mobile phase consisted of a mixture of 5% formic acid in water (eluent A) and 5% formic acid in acetonitrile (eluent B) and was applied at 20°C and a flow rate of 0.5 mL min^−1^. The following gradient was used to elute the FA: 0 min–100% A, 15 min–100% B. FA was detected by a DAD at 320 nm. FA concentrations were calculated using a calibration curve linear in the range from 0.001 mg mL^−1^ to 0.05 mg mL^−1^.

Toc was analysed on a Hitachi system (LaChrom Elite, VWR/Hitachi, Darmstadt, Germany) applying the following method: 10 *μ*L was injected onto the column (C30 reversed phase column, 5 *μ*m, 250 x 4.6 mm, YMC Europe, Dinslaken, Germany). The sample was eluted with a flow rate of 1 mL min^−1^ at 27°C using the following gradient: 0 min–81% methanol, 15% methyl t-butyl ether (MTBE), 4% water; 10 min–72.4% methanol, 23.6% MTBE, 4% water. Toc was detected by a DAD at 292 nm. Toc concentrations were calculated using a calibration curve that was linear in the range from 0.005 mg mL^−1^ to 0.05 mg mL^−1^.

### Radical scavenging activity

SLN suspensions were diluted 20-fold with acetic acid buffer solution. A second 2-fold dilution step was applied by adding 500 *μ*L of 2 mM Fremy’s salt in acetic acid buffer solution to an equal volume of the diluted SLN suspension yielding final antioxidant and Fremy’s salt concentrations of 0.05 mM to 0.3 mM and 1 mM, respectively. The mixtures were allowed to react for 30 min at 20°C in the dark. The samples were filled into micropipettes which were then placed in 0.5 mm quartz tubes. Electron paramagnetic resonance spectroscopy (EPR) measurements were carried out using an e-scan system (Bruker, Rheinstetten, Germany) with the following settings: center field 3482 G, sweep width 150 G, microwave frequency 9.81 GHz, microwave amplitude 1.83 G, conversion time 10.24 ms. The spectrum of Fremy’s radical was integrated twice using the software WinEPR (Bruker, Rheinstetten, Germany) [17]. The peak areas were used to calculate the remaining Fremy’s salt concentrations using a calibration curve. A second set of measurements was carried out applying different dilution steps to the SLN suspensions to reach final FA or Toc concentrations of 0.13 mM. The measurements were carried out under the same conditions as described above. Two independent measurements were taken for each sample yielding a total of 6 individual values for each concentration and antioxidant. Linear regression of the concentration of reduced Fremy’s radical vs. antioxidant concentration was performed using the software SigmaPlot 12.3 and slopes and standard errors were calculated.

### X-ray diffraction (XRD) measurements

The samples were concentrated stepwise using centrifugal ultrafiltration devices (VivaSpin 2, 300 kDa, Sartorius Stedim Biotech, Göttingen, Germany) at 4000 g for up to 2 h to increase the total lipid content. This step did not affect the crystal structure of the samples as revealed by supporting DSC measurements. XRD measurements were performed on a Bruker D8 Advance (Bruker AXS, Karlsruhe, Germany). About 600 *μ*L of the concentrated samples was filled into the round indentation (25 mm diameter, 1 mm depth) of a PMMA sample holder and was measured in liquid at ambient conditions. The measurements were carried out using Cu K*α*1,2 radiation (λ = 0.15418 nm). Data were collected in the 2*θ* range of 14° to 30°, with a step width of 0.24° 2*θ* and 252 s per step applying a position sensitive detector (PSD) Lynxeye. The background was corrected by subtracting a blank. Differences in the filling heights in the sample holder resulted in shifts of the diffractograms due to different curvatures of the liquid surface (height error). These shifts were corrected by aligning the first peaks in the diffractograms with the first peak measured in a dry bulk sample of tristearin. d-values were calculated from the local maxima of the 2*θ*-diffractogram by applying Bragg’s-Law: *n*λ = 2*dsin*(*θ*).

### DSC measurements

The melting behaviour of blank SLN, FA-SLN and Toc-SLN was analysed by differential scanning calorimetry (DSC, Q2000, TA Instruments, New Castle, USA). Approx. 10 mg of SLN suspension was transferred into aluminium pans (Tzero) which were then hermetically sealed. Samples were equilibrated at 25°C for 5 min and then heated to 80°C at a rate of 1 K min^−1^ or 10 K min^−1^. The thermograms were evaluated using the software TA Universal Analysis 2000 (TA Instruments, Eschborn, Germany) with respect to peak maximum temperature and peak areas, i.e. melting enthalpy. An empty pan was used as a reference. Two independent measurements were taken for each sample yielding a total of 6 individual values for each particle type and heating rate.

### Statistical analysis

All samples were prepared in triplicates and measurements were performed two to six times as indicated in the respective sections. Results are presented as means with standard deviations. Samples were compared by a one sided ANOVA test followed by the Holm-Sidak post-hoc test using the software SigmaPlot 12.3. Results were considered statistically different with p < 0.05.

## Results and discussion

### Particle size distribution and zeta potential

**Fig 1:**
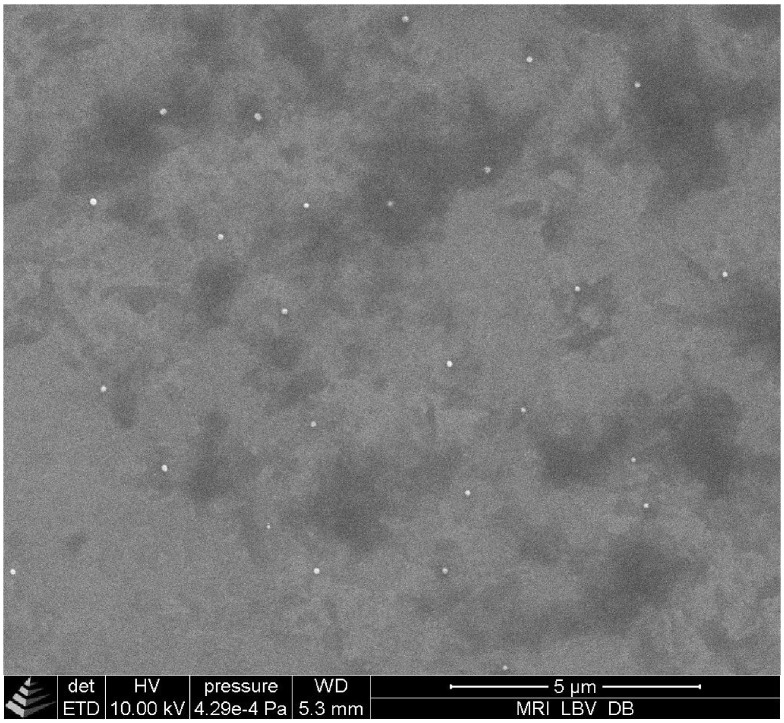
Representative scanning electron microscopy image of FA-SLN. The particle size of the depicted particles is in the range of 100 to 130 nm.

**Fig 2:**
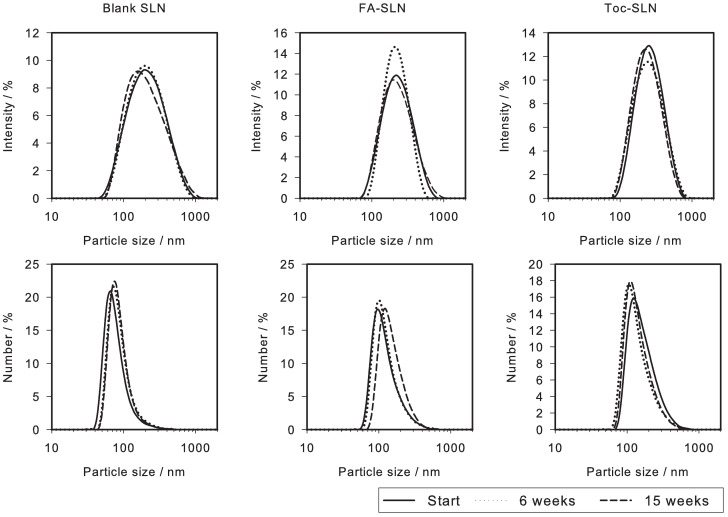
Representative intensity and number based particle size distributions of blank SLN, FA-SLN and Toc-SLN at times zero, 6 weeks and 15 weeks.

Scanning electron micrographs revealed nearly spherical or ellipsoid particles of 100 nm to 130 nm (Fig 1). The size distributions of the SLN as determined by DLS presented a PdI of about 0.2, which indicates the existence of a narrow to moderate polydisperse population(Fig 2). The average particle diameter (z-ave) was in the range of 180 nm to 200 nm. The loading of SLN with FA or Toc did not lead to any substantial changes in particle size distributions (Table 1, Fig 2). Antioxidant concentration dependent variations in particle size were not expected because the appropriate amounts of tristearate were replaced by the respective antioxidant. In the literature, decreased [9, 12, 19], increased [20–23] and unaffected [8] particle sizes were observed after loading SLN with different bioactive compounds. These studies exemplify that the incorporation of compounds into SLN does not necessarily affect the particle size. Furthermore, the PdI reported in those studies were in the range of 0.05 to 0.31. Hence, the PdI of the particle size distributions of the present study are well within the range of values reported in the literature. The non-spherical shape of SLN as depicted in Fig 1 has been reported earlier and is an effect of the crystal structure of the matrix lipid [16]. This should be taken into account when interpreting DLS data. Because underlying algorithms assume spherical particles, DLS might not be sufficiently sensitive to detect small changes or differences in particle sizes of platelet shaped particles like SLN.

**Table 1:** Z-average, polydispersity index (PdI) and zeta potential of SLN directly after preparation and after 6 and 15 weeks of storage, n = 3.

AOx^a^	AOx concn. mg g^−1^	Z-Average / nm			PDI			ZP / mV		
		Start	6 weeks	15 weeks	Start	6 weeks	15 weeks	Start	6 weeks	15 weeks
Blank	0	188 ± 18	187 ± 14	180 ± 14	0.232 ± 0.042	0.232 ± 0.033	0.226 ± 0.021	-32.9 ± 4.1	-34.2 ± 0.5	-37.0 ± 1.5
FA	0.55	205 ± 35	183 ± 7	179 ± 6	0.288 ± 0.103	0.205 ± 0.014	0.202 ± 0.014	-25.8 ± 1.4	-35.2 ± 1.7	-38.0 ± 2.3
	1.16	172 ± 23	180 ± 17	185 ± 8	0.243 ± 0.035	0.226 ± 0.026	0.205 ± 0.009	-29.6 ± 5.0	-36.0 ± 1.2	-39.8 ± 0.5
	1.67	188 ± 20	187 ± 12	183 ± 11	0.207 ± 0.006	0.190 ± 0.024	0.196 ± 0.018	-26.7 ± 1.0	-35.7 ± 0.5	-39.0 ± 0.4
	2.19	184 ± 5	180 ± 4	179 ± 4	0.193 ± 0.018	0.184 ± 0.023	0.192 ± 0.012	-28.5 ± 5.9	-35.4 ± 0.3	-39.0 ± 0.9
	2.80	177 ± 36	184 ± 29	180 ± 30	0.208 ± 0.026	0.183 ± 0.039	0.181 ± 0.044	-28.0 ± 0.9	-35.2 ± 1.2	-38.5 ± 0.4
Toc	0.56	213 ± 26	205 ± 21	202 ± 22	0.229 ± 0.01	0.201 ± 0.005	0.193 ± 0.028	-28.6 ± 3.6	-36.8 ± 0.5	-38.7 ± 1.4
	1.16	204 ± 11	193 ± 6	188 ± 6	0.202 ± 0.02	0.183 ± 0.019	0.196 ± 0.008	-25.6 ± 0.7	-35.9 ± 0.7	-38.2 ± 0.6
	1.67	202 ± 4	196 ± 1	191 ± 1	0.199 ± 0.008	0.188 ± 0.025	0.172 ± 0.012	-30.9 ± 2.9	-35.2 ± 0.9	-39.1 ± 0.6
	2.19	193 ± 4	190 ± 2	185 ± 1	0.193 ± 0.014	0.166 ± 0.009	0.175 ± 0.022	-30.2 ± 3.8	-35.5 ± 0.0	-39.0 ± 1.0
	2.80	216 ± 19	205 ± 36	248 ± 77	0.191 ± 0.021	0.195 ± 0.012	0.255 ± 0.164	-30.9 ± 4.0	-37.2 ± 1.0	-42.9 ± 5.6

^a^ Antioxidant (FA or Toc)

Blank and loaded SLN were stable with respect to the particle size distribution over 15 weeks of storage. Neither the intensity nor the number based particle size distributions revealed the occurrence of a second population as might be possible by particle aggregation (Table 1, Fig 2). The high physical stability of SLN at room temperature up to three years has already been reported in the literature [2] and is one reason for the high interest in this kind of particles. However, the stability may be impaired by uncontrolled crystallisation behaviour leading to the gelation of the SLN suspension [24]. The stability of SLN was shown to be temperature dependent with elevated temperatures leading to increased particle sizes [20] or accelerated gelation [25]. It was further reported that the stability of SLN was sensitive to the pH value and ionic strength of the surrounding medium [26]. Thus, in the present study, the aqueous phase of the initial suspension (0.1 M sodium acetate buffer solution, pH 5.0) was suitable to maintain a long term physical stability.

The zeta potential (ZP) of the SLN as a measure for the surface charge was investigated to gain more insight into the surface properties related to the loading with antioxidants. The ZP of the SLN in the present study was in the range of −25 mV to −43 mV (Table 1). It was independent of the loading with antioxidants, but increased over time in FA or Toc loaded SLN. In FA-SLN the ZP increased by about 10 mV during the first 6 weeks of storage and another 5 mV during 9 more weeks. The ZP of Toc-SLN also increased mainly within the first 6 weeks of storage and in total 10 mV over 15 weeks of storage. It was reported in the literature that the ZP of SLN remained unchanged upon loading with quercetin [22, 23] or that the ZP of Toc-SLN was independent of the tocopherol content in another study [5]. However, when particles were prepared under optimised conditions, Toc-loaded SLN had reduced ZP compared to unloaded SLN [5]. FA-loaded SLN had about 5 mV higher ZP than the blank SLN [8]. In another study the ZP of umbelliferone loaded SLN was increased by 40 mV compared to blank SLN although umbelliferone is a non-ionic molecule [19]. Decreased ZP was observed when loading SLN with the non-ionic resveratrol or co-enzyme Q10 [12, 21]. The ZP would be expected to be sensitive to the presence of ionic compounds at the particle surface. FA is negatively charged at pH 5 (*pK*_*a*_ = 4.5) [27]. Because the change in ZP was independent of the FA content in SLN in the present study, it is suggested that either the effect of FA near the surface was below the detection limit or that the charges were buried in the lipid matrix or the emulsifier layer. Tocopherol as a non-ionic compound was not expected to cause significant changes in the zeta potential. Nevertheless also the ZP of Toc-SLN increased over time. From this survey and own data it is concluded that the impact of bioactive compounds on the ZP of SLN depends more on the molecular structure than the electric charge of the compound. Because the ZP and its change over time was independent of the type or concentration of antioxidants in the present study it is suggested that structural re-arrangements near the surface caused the change in ZP.

### Recovery and stability of antioxidants

Directly after preparation the recovery of FA was 87.7% ± 1.7% irrespective of the initial FA content in the SLN (Table 2). After 15 weeks of storage the average recovery decreased to 82.8 ± 2.6%. In Toc-SLN the average recovery decreased from 77.4 ± 6.7% after preparation to 68.2 ± 6.5% after 15 weeks of storage and decreased with increasing initial Toc concentration.

**Table 2:** Recovery of FA and Toc from SLN directly after preparation and after 15 weeks of storage, n = 3.

Aox	conc. mg g^−1^	Recovery / %	
		Start	15 weeks
FA	0.56	87.5 ± 2.3	80.6 ± 2.5
	1.16	87.8 ± 3.1	82.4 ± 3.9
	1.67	88.6 ± 1.8	84.7 ± 0.9
	2.19	87.6 ± 1.3	83.7 ± 0.8
	2.80	86.9 ± 2.1	82.5 ± 3.6
Toc	0.56	76.9 ± 11.5	79.3 ± 3.7
	1.16	80.9 ± 8.6	69.0 ± 2.9
	1.67	78.6 ± 1.2	65.4 ± 1.8
	2.19	74.6 ± 3.3	63.4 ± 1.0
	2.80	75.6 ± 1.1	63.9 ± 2.8

The initial loss of FA and Toc may have resulted from the elevated temperatures during particle preparation. To avoid FA or Toc degradation, the respective antioxidant was added after homogenisation but before the cooling step. However, to guarantee full dissolution of the respective compound and its incorporation into the SLN, the hot suspension was stirred for at least 10 min before the Tween 20 solution was added. Despite the partial degradation of both antioxidants during particle preparation, they were fairly stable at room temperature. The recovery rates found in the present study are well within the range of results reported in the literature. E.g. the recovery of Toc after incorporation into SLN was 75% and increased with increasing Toc content, while lower recoveries of Toc (66%) were reported in samples stored at 20°C for three weeks [5]. In another study, SLN encapsulated Toc was stable for three months of storage [20]. The influence of the emulsifiers used to stabilise the SLN on the storage stability of SLN encapsulated *β*-carotene was demonstrated using lecithins and Tweens. In that study the stability of *β*-carotene during storage decreased in the order Tween 80 < Tween 60 < high melting lecithin [4]. Hence, the mixture of high melting lecithin and Tween 20 used in the present study represents a suitable formulation with respect to both, chemical stability of the encapsulated compounds and physical stability of the SLN.

### Encapsulation rates

**Fig 3:**
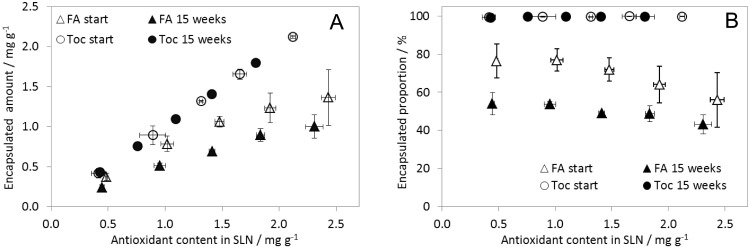
Encapsulation efficiency of FA and Toc in SLN. A) Encapsulated absolute amounts and B) encapsulation rates. Total concentrations on the x-axis refer to the extracted amounts of FA or Toc. The dataset is available in S1 Dataset.

The initial encapsulation efficiency of FA in SLN decreased with increasing total FA content from about 80% to 60% which translates into payloads of 0.4 mg g^−1^ to 1.3 mg g^−1^ (Fig 3). During storage, FA was partially released into the aqueous phase or micellar pseudophase resulting in encapsulated proportions between 55% and 45%. The release was concentration dependent and increased with total FA concentrations. Similar payloads and encapsulation rates for FA in SLN were reported earlier [8]. These results are in line with the partitioning behaviour of phenolic compounds in o/w emulsions. Encapsulation efficiencies of FA in 10% corn oil in water emulsions stabilised by a non-ionic emulsifier were in the range of 60% [27]. Before cooling, the lipid nanoparticles are hot nanoemulsions so that similar partitioning behaviour is expected as in o/w emulsions. I.e. the partitioning of a compound between the aqueous phase, emulsifier pseudophase and lipid phase depends on solubilisation capacities and interactions with emulsifier molecules [27, 28]. The higher encapsulation rates in the present study may have resulted from the increased surface area of the SLN compared to the oil droplets in the emulsions of the mentioned study (approx. 1 *μ*m).

Non-encapsulated tocopherol was below the limit of quantification at each point, i.e. encapsulation efficiencies were close to 100% both at the beginning of the experiment and after 15 weeks of storage. This is in good agreement with earlier studies with Toc-SLN of different composition. Toc-SLN were prepared for the use in sunscreen with a payload of 0.5% w/w [29]. In another study, the payload of 1.1% Toc was reported to be optimal with respect to particle size and ZP of the SLN and Toc recovery [5]. Also other lipophilic compounds were encapsulated in SLN with near to 100% encapsulation efficiencies like vitamin A [7], rosmarinic acid [6], quercetin (93%, payload of 0.6%) [22] or co-enzyme Q10 (89%) [12]. Stearyl ferulate as lipid matrix and the application of washing steps led to lower encapsulation efficiencies (59%) [10]. Those SLN consisted of stearyl ferulate instead of a triglyceride, and the particles were washed several times. Tocopherol may have been solubilised by emulsifier micelles and may therefore have been lost during the washing steps.

With both FA and Toc the encapsulation rates were comparable to literature data reporting on SLN or emulsions with a triglyceride lipid matrix. The higher encapsulation rate of Toc can be explained by its higher lipophilicity.

### Radical scavenging activity

**Fig 4:**
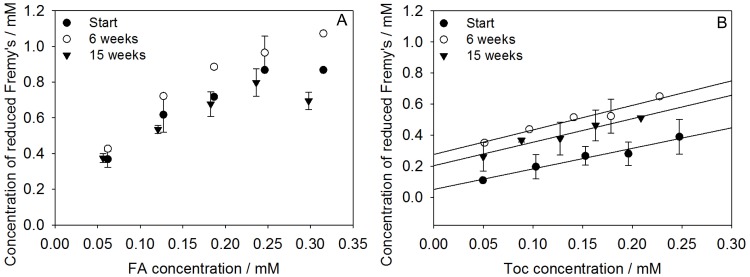
Radical scavenging activity of FA-SLN (A) and Toc-SLN (B). Samples were analyzsed directly after preparation, 6 weeks and 15 weeks of storage. SLN were diluted 40-fold resulting in antioxidant concentrations given on the x-axis. The dataset is available in S2 Dataset.

The radical scavenging activity of the encapsulated antioxidants was followed by the reduction of Fremy’s salt using EPR spectroscopy. To achieve suitable reaction conditions the SLN suspensions were diluted 40-fold and allowed to react with 1 mM Fremy’s salt. Preliminary experiments revealed that the activity of FA or Toc depended on the total FA or Toc concentration in the reaction medium but not on the dilution factor (data not shown). In freshly prepared FA-SLN a near linear reduction of Fremy’s salt with increasing FA concentration was observed (Fig 4a). A plateau was reached where Fremy’s salt was completely reduced. Therefore, the region above 1.7 mg g^−1^ could not be used to interpret time-dependent changes in the radical scavenging activity of FA-SLN. Linear relationships between Toc concentrations and reduced Fremy’s salt were observed after each storage period (Fig 4b). The slopes of the linear regression curves were 1.32±0.14, 1.51±0.20 and 1.58±0.18 at the beginning and after 6 and 15 weeks, respectively.

The reactivity of antioxidants in dispersed systems depends on the location of both radical and antioxidant in various ways. 1) The reactants must be in close proximity. The emulsifier layer represents a physical barrier if the solubilisation properties of antioxidants and radicals differ markedly [17]. 2) The location represents a chemical environment that can be more or less favourable for the reaction. The local pH value in a micellar system has been demonstrated to affect the radical scavenging activity of FA [27]. 3) The mobility of the reactants affects their diffusion and thus the likelyhood of a contact. This was demonstrated to affect the radical scavenging activity of Trolox in o/w emulsions [30] or Tocopherol in liposomes [31]. In an earlier study it was shown that Fremy’s salt was located in the water rich palisade layer in Brij micelles [17]. It is suggested that Fremy’s radical was located in a region of the SLN surface which is structurally similar to the palisade layer of non-ionic micelles. It is suggested that time dependent structural rearrangements affected the accessibility of Fremy’s salt for Toc but not for FA.

### Crystal structure and melting behaviour

In the present study, solidification of the lipid particles occurred during the very fast cooling of the suspension to room temperature (approx. 40 K min^−1^). The crystalline state of the SLN was studied by XRD.

**Fig 5:**
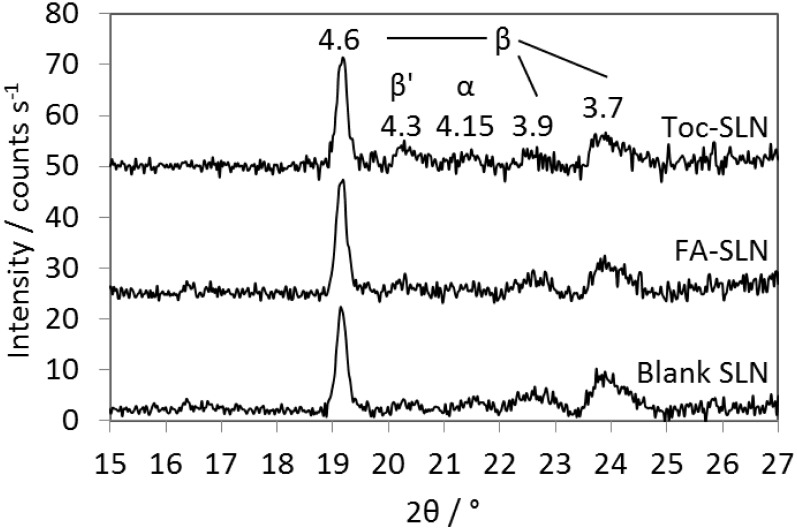
XRD patterns of blank SLN, FA-SLN and Toc-SLN. Peaks were assigned to modifications typically described in the literature. XRD patterns were scaled to the intensity of the first peak.

Freshly prepared blank SLN, FA-SLN and Toc-SLN yielded similar XRD patterns with reflections at 2*θ* 19.1°, 20.2°, 21.5°, 22.6°, and 23.8° corresponding to d-values of 4.6 Å, 4.3 Å, 4.1 Å, 3.9 Å and 3.7 Å, respectively (Fig 5). These distances are usually ascribed to the short spacings of *α*, *β*′ and *β* modifications of triglycerides [32]. The co-existence of different polymorphs and the dependence of their formation on factors like agitation, tempering and the presence of an emulsifier has been demonstrated e.g. in hydrogenated cotton seed oil [33]. In the same study, shifts in the short spacings of *β*′ polymorphs have also been reported. Which polymorphs were formed in the present study cannot be unambiguously clarified from the data and would need further investigation. However, the reflections at 21.5° and 20.2° were very small indicating that the amount of the respective structures were relatively low. Loading the SLN with FA or Toc did not interfere with the crystallisation of the tristearin as revealed by the XRD measurements.

The melting behaviour of blank and loaded SLN was studied using DSC. Melting enthalpies were in the range of 5.4 J g^−1^ to 7.7 J g^−1^ and were inversely proportional to the content of FA or Toc, i.e. proportional to the triglyceride content of the SLN. The melting enthalpies did not change over time, indicating that the crystallinity obtained during cooling remained constant over a storage period of 15 weeks at 25°C. This result does not match with the ones of other studies were the addition of Toc and emulsifiers to the lipid matrix led to a reduced crystallinity [5]. However, formulation and process parameters have a strong impact on the crystallisation of the lipid matrix [34].

**Fig 6:**
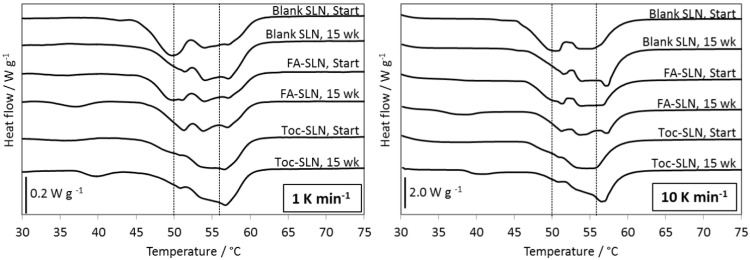
Representative melting curves of loaded and unloaded SLN. Samples were analysed directly after preparation and after 15 weeks of storage recorded applying heating rates of 1 K min^−1^ or 10 K min^−1^. FA or Toc contents in the SLN were 2.8 mg g^−1^. Vertical lines at 50°C and 56°C serve as guides to the eye.

The melting range of both blank and loaded SLN was relatively broad, with onset-offset differences of about 20°C (Fig 6). Unloaded SLN heated at 1 K min^−1^ presented endothermic peaks at 50°C, 54°C and 57°C and an exothermic peak at 52°C.

Various authors observed that SLN prepared with monoacid triglycerides and particle size below 200 nm, as is here the case, show a particular size dependent melting behaviour with multiple peaks which are not due to polymorphism but to the melting of particles with different thicknesses [6, 35–37].

The melting behaviour of triglycerides may undergo more or less important modifications due to the presence of other components, such as surfactants or encapsulated compounds. According to Oh and co-workers the addition of up to 5% of sucrose stearate to tristearin exhibited little effect on the *α* to *β* transition of tristearin [38]. Other authors observed that the presence of 5% of sucrose monostearate hindered the *β*′ to *β* transformation [39]. Working with Tween 20 in concentrations of up to 6% in the suspension, Helgason and co-workers detected an increase of the number of peaks and a shift to lower temperatures in the melting transition of tripalmitin as the surfactant concentration in the SLN suspensions increased [40]. In our study however, the most important modification observed was the broadening of the melting range. Differences in the melting behaviour of loaded and unloaded SLN have also been reported, e.g. onset, offset and melting temperature were reduced in Toc-SLN as compared to blank SLN [5]. However, according to Bunjes et al. as generally the amount of incorporated compound is low in relation to the lipid matrix, no pronounced effects are expected [37].

Time and antioxidant concentration dependent changes in the melting behaviour of blank and loaded SLN were studied using low (1 K min^−1^) and high (10 K min^−1^) heating rates in DSC measurements. During DSC measurements with low heating rates solid-solid transitions prevail, while with high heating rates melt-mediated transitions do [41]. When heated at 1 K min^−1^ all SLN showed three distinct sub-peaks both at the beginning of the experiment and after 15 weeks of storage (Fig 6). When heated at 10 K min^−1^ the second peak was initially very broad and the third peak appeared only after 6 or 15 weeks of storage. The different melting behaviour observed coincides with studies were melt mediated polymorphic transitions occurred during the measurements at low heating rates [32]. In this case the melting curves may not reflect the actual state of the solid lipid matrix. The appearance of a more intense peak at around 56°C after storage indicates that during storage either the tristearin underwent re-structuring resulting in an increased proportion of the stable *β* modification or that a fraction of particles increased in size. The latter was not reflected by the DLS measurements. Furthermore, the reduction of the peak at around 50°C after storage would support the assumption that the first and third peaks observed could mainly be ascribed to the (*α*) and (*β*) modifications of tristearin.

**Fig 7:**
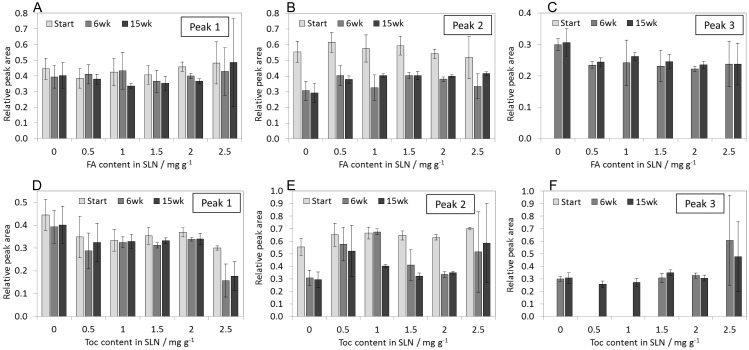
Proportions of peak 1, peak 2 and peak 3 in melting curves of SLN containing different amounts of FA (A-C) or Toc (D-F). The dataset is available in S3 Dataset.

Although the general trend of the melting profiles was similar for all three SLN types, the proportion of the observed peaks was different. This was used in an attempt to quantitatively describe the observed time and concentration dependent changes of the melting behaviour. Therefore the areas of the sub-peaks obtained with the high heating rate were integrated and related to the total peak areas (Fig 7).

The assignment of peaks to certain thermal events in complex samples such as the SLN studied here, is challenging. Therefore the peaks will not be assigned to any polymorphic form in particular and further in this paper we will refer to peaks 1, 2 and 3.

The relative area of peak 1 was in the range of 0.4 for blank SLN and for FA-SLN irrespective of the total FA content in SLN or storage time. For Toc-SLN peak 1 showed a relative area in the range of 0.35 over the entire range of concentrations, while only in SLN with the highest Toc content, 2.5 mg g^−1^ Toc, the relative area of this peak decreased significantly after 6 weeks of storage and then remained unchanged up to week 15. The relative area of peak 2 was in the range of 0.5 to 0.6 for freshly prepared blank SLN and FA-SLN, irrespective of the FA content. After 6 weeks the contribution of this peak was reduced almost to the half in blank SLN and in FA-SLN, remaining unchanged up to 15 weeks. For freshly prepared Toc-SLN the contribution of peak 2 was slightly higher than for the previous samples, decreasing slowly along the storage period studied.

Due to the broad melting range of all samples, peak 3 could not be evaluated for freshly prepared samples. After 6 weeks peak 3 presented a relative area of approx. 0.3 for blank SLN and FA-SLN, remaining unchanged for the rest of the storage period. In the case of Toc-SLN peak 3 was evident only after 15 weeks for SLN with low Toc content, while for Toc-SLN containing 1.5 mg g^−1^ or more Toc the peak was detected already after 6 weeks of storage.

As a result of this evaluation, it can be concluded that the thermal behaviour of blank SLN and FA-SLN was very similar both just after preparation and along storage. Furthermore, while these SLN suffered modifications within 6 weeks of storage irrespective of the FA concentration, changes in Toc-SLN occurred more slowly and were concentration dependent. These results suggest that the FA was not incorporated in the lipid matrix while Toc may have been incorporated or at least been located in close proximity of the lipid matrix.

### Proposed location and mechanism

The radical scavenging activities of FA-SLN remained unchanged during the storage period of 15 weeks whereas the activity of Toc-SLN showed time dependent changes. These changes could neither be explained by changes in encapsulation rates or degradation of the antioxidants during storage. Hence, it is suggested, that changes in the location of Toc and hence the chemical environment and / or accessibility to Fremy’s salt was responsible for the increased activity after 6 and 15 weeks of storage. Such structural changes could be reflected by the changes in the crystal structure and melting behaviour.

Initially the lipid matrices of unloaded and loaded SLN contained, if at all, low amounts of *α* and *β*′ polymorphs. The presence of different polymorphs cannot be unambiguously confirmed. However, time dependent changes in the melting behaviour reflect structural changes during storage. If they were related to polymorphic transitions the following point should be addressed: Packing requirements for the *β* polymorph are more stringend than for the *α* or *β*′ polymorph [42]. Thus, the *α* or *β*′ polymorphs allow for a better encapsulation or incorporation of encapsulated molecules. The crystallisation of the lipid matrix, or the *α* or *β*′-to-*β* transition results in the expulsion of an encapsulated compound [7, 43–45]. In the present study changes in the melting behaviour were retarded in a concentration dependent manner in Toc-SLN. Time dependent changes in FA-SLN were similar to blank SLN. Based on these observations we hypothesise that the different molecular structures and locations of FA and Toc caused the differences in the melting behaviour. The location of encapsulated compounds, i.e. how much they would be affected by polymorphic transitions, depends on their molecular structure and solubilisation properties. With EPR spin probes it was demonstrated that lipophilic compounds were located in the lipid matrix whereas more hydrophilic compounds were located at the surface of the SLN, i.e. in the headgroup region of the emulsifiers [13–15]. In o/w emulsions it was found that, depending on the type of oil phase, emulsifier type and concentration, up to 95% of tocopherol were located in the emulsifier layer of oil droplets rather than in the droplet core [46]. Hence, we propose that Toc was located near the lipid matrix of the SLN, probably in the tail region of the emulsifiers. In contrast, FA is suggested to be located in the outer headgroup regions of the emulsifier layer at the surface of the SLN. This would explain the stronger impact of Toc on the (crystal) structure of the lipid matrix. This also implies that Toc was affected more strongly by structural re-arrangements than FA, because it was in closer contact to the lipid phase.

The impact of Toc on the melting behaviour could either be direct or emulsifier mediated: If Toc is located in the lipid matrix it could directly affect the melting behaviour and would directly be influenced by structural re-arrangements. An emulsifier mediated mechanism would be based on irregularities in the emulsifier chain region caused by tocopherol that in turn affect the melting behaviour and / or the changed arrangement of emulsifiers upon structural re-arrangements of the lipid matrix would affected Toc. Both mechanisms seem possible and their clarification needs further investigation.

## Summary and conclusions

It was possible to prepare FA and Toc loaded SLN with payloads up to 2.5 mg/g without significant effects on particle size distribution and zeta potential. The melting behaviour was affected by the type and concentration of the antioxidants. Whereas the effect of FA was negligible, the presence of different amounts of Toc significantly affected time dependent changes in the melting behaviour. Structural re-arrangements and / or the possible expulsion of Toc from the SLN increased its radicals scavenging activity. While the activity of FA-SLN was stable over the entire storage period, the activity of Toc-SLN increased over time. Overall, the SLN and the antioxidants showed good stability and radical scavenging activity during 15 weeks of storage at room temperature and may thus be suitable as additives for food systems were a gradual release of the active compound could be beneficial.

## Supporting information

S1 Dataset Absolute encapsulated amounts and encapsulation rates of FA and Toc after times zero, 6 weeks and 15 weeks as determined by ultrafiltration and HPLC analysis of the filtrates. (XLSX)

S2 Dataset Proportions of peak, peak 2 and peak 3 in melting curves of SLN containing different amounts of FA or Toc. (XLSX)

S3 Dataset Radical scavenging activities of FA-SLN and Toc-SLN at times zero, 6 weeks and 15 weeks of storage. FA and Toc concentrations resulted from 40-fold dilution in the reaction caps.
(XLSX)
